# Physicochemical Evaluation of Personal Care Products Developed with *Chondrus crispus* Fractions Processed by Ecofriendly Methodologies

**DOI:** 10.3390/md20110695

**Published:** 2022-11-06

**Authors:** Lucía López-Hortas, María Dolores Torres, Elena Falqué, Herminia Domínguez

**Affiliations:** 1 CINBIO (Centro de Investigaciones Biomédicas), Chemical Engineering Department, University of Vigo (Campus Ourense), Polytechnic Building, As Lagoas, 32004 Ourense, Spain; 2 Department of Analytical Chemistry, Faculty of Sciences, University of Vigo, As Lagoas s/n, 32004 Ourense, Spain

**Keywords:** body milk, body oil, shampoo, body scrub, autohydrolysis, microwave-assisted extraction, ultrasound-assisted extraction, microwave hydrodiffusion and gravity technology

## Abstract

Novel personal care products are necessary to cope with the growing market demand for sustainable green products. In this context, this work deals with the formulation and fundamental physicochemical and rheological characterization of different natural personal care products using bioactive fractions from *Chondrus crispus* red macroalgae extracted under optimized green conditions. Body milks, body oils and shampoos were supplemented with soluble extracts with antioxidant features recovered after hydrothermal (200 °C) and microwave (170 °C)- and ultrasound (80 °C)-assisted extraction of the red macroalgae used as raw material. Formulated products were also compared with those prepared using (±)-α-tocopherol and butylhydroxytoluene standards. Body scrubs were formulated with the remaining solids (<2.25%) after microwave hydrodiffusion and gravidity treatment of the macroalgae. Results indicated that selected extracts provided personal care products with similar or even better physicochemical, color and viscous features than those supplemented with (±)-α-tocopherol or butylhydroxytoluene commercial antioxidants. Rheological profiles indicated that it is possible to develop personal care products with adequate viscous behavior (10^2^–10^5^ mPa s, at 1 s^−1^), comparable with their synthetic counterparts. To conclude, the addition of antioxidant extracts led to lower apparent viscosity values suggesting an advantage from the skin applicability point of view, jointly with the absence of both the hysteresis phenomenon and water syneresis of the proposed formulations.

## 1. Introduction

Consumers are increasingly demanding green and ecofriendly products in the fields of food, cosmetics and personal care products [1]. The use of renewable and sustainable raw materials, containing high-added valuable compounds, with green extractive strategies responds to a growing market interest encouraged by the environmental concerns of consumers and the industry requirements of ensuring the availability of resources for their production [2]. The cosmetics industry is a key industrial sector, where marine cosmetics, also known as phycocosmetics, are an economic reality [3]. Even though plant-derived extracts are commonly among the principal additives of natural cosmetics [4], bioactive compounds from algae have a relevant application in this field. These marine extracts have the capacity to mitigate the generation of process-generated unwanted chemicals, as well as the health benefits that they provide, when compared with the potential health risks that the use of synthetic antioxidants could produce [5]. Several beneficial metabolites can be obtained from algae, such as antioxidants, mycosporine-like amino acids, carotenoids, pigments or sulfated polysaccharides [4].

Various algae sources are currently widely used in skincare cosmetics for several purposes, such as moisturizers, texture-enhancing agents, antiwrinkle agents or sunscreen [1,6]. Red algae are rich sources of natural polysaccharides with promising features to be used in cosmeceutical industries as a whitening agent [6]. Red algae can feature antioxidative, photoprotective, anti-inflammatory and antimelanogenic activities [5]. Their antioxidative activity was correlated with the presence of polyphenols by many researchers [3].

Extracts from red macroalgae *Chondrus crispus* Stackhouse 1797 could offer valuable ingredients for personal care products since it possesses fractions with sunscreen and antiaging potential [7]. Its extracts, mainly carrageenan, can act as thickening, water-binding and stabilizing agents, as well as possess different biological activities that make it adequate as an ingredient in personal care products [8], and consequently give to the cosmetic market an interesting perspective of novel ingredients to develop enhanced formulations to combat the inevitable skin aging. Further research should be performed by assessing its potential through in vitro studies, thus paving way for red algae to be incorporated into suitable formulations for both cosmetic and treatment use [1,8].

Antioxidant biomolecules can be used in numerous novel applications (e.g., shampoos, cosmetic creams, body oils and gelled matrices, among others), which is indicative of the technological relevance of these bioactive compounds [7]. The physicochemical and phytochemical characterization of the bioactive extracts is critically relevant in order to select the most adequate final application, as well as to determine the rheology of the developed formulations [9,10]. During the processing of personal care products, the presence of bioactive extracts must be carefully taken into account to avoid losses in their healthy features. The monitoring of the viscosity of the formulated matrices during manufacturing and in the final product is critically relevant for the right selection of the processing conditions and to enhance the quality and shelf life of the end product [9,11]. The challenge is to obtain functional matrices with tailored rheological properties suitable for personal care applications developed in a sustainable way.

In this context, the main aim of this work is to assess the feasibility of formulating different natural personal care products supplemented with marine extracts recovered using green extraction technologies, including the fundamental physicochemical and rheological characterization.

## 2. Results

**Figure 1:**
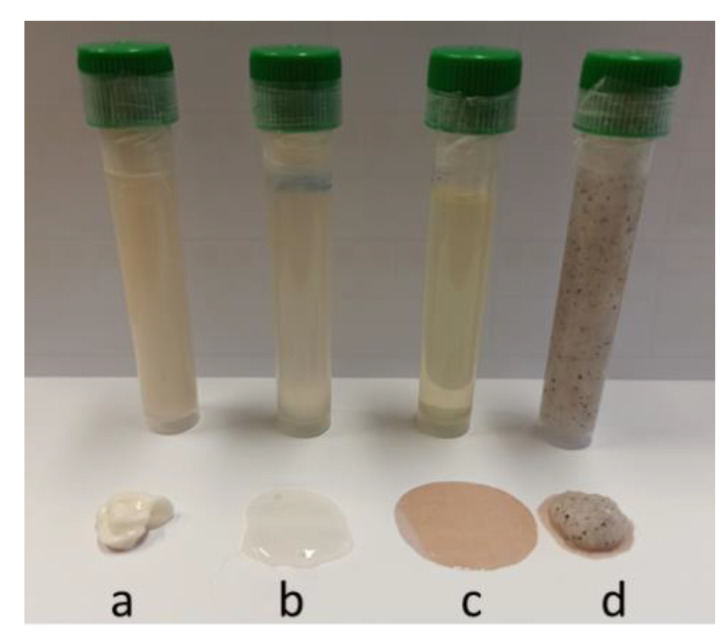
Personal care products produced from *C. crispus* extracts or dried macroalgae: (**a**) body milk; (**b**) shampoo; (**c**) body oil; (**d**) body scrub.

Three different personal care products (body milk, body oil and shampoo) were elaborated from *Chondrus crispus* extracts obtained by applying autohydrolysis (AH), microwave-assisted extraction (MAE) and ultrasound-assisted extraction (UAE) technologies, and the body scrub was formulated from the dried red macroalgae by microwave hydrodiffusion and gravity (MHG) (Figure 1).

**Figure 2:**
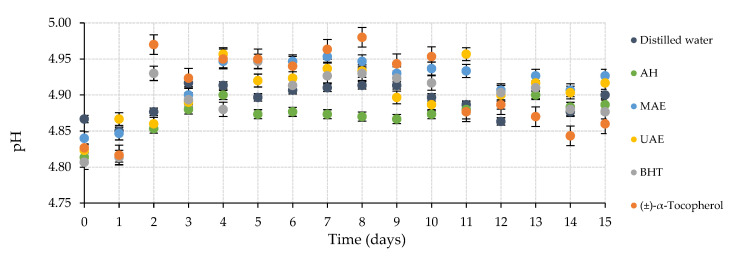
Compilation of pH values in accelerated oxidation assay at 50 °C during 15 days of the body milk samples elaborated with different *C. crispus* extracts. AH: Autohydrolysis; MAE: Microwave-assisted extraction; UAE: Ultrasound-assisted extraction; BHT: Butylated hydroxytoluene.

The evaluation of the potential use of *Chondrus crispus* extracts and dried macroalgae as cosmetic ingredients could be determined through their stability study by an accelerated oxidation assay at 50 °C for 15 days. The results of this test on body milk samples are shown in Figure 2. The obtained pH values fluctuated between 4.81 and 4.98. This reduced difference reflected the limited experimented variation in this chemical parameter under the work conditions mentioned above. Overall, samples elaborated with autohydrolysis (AH) extracts maintained lower values, whereas personal care products enriched with (±)-α-tocopherol and microwave-assisted extraction (MAE) presented higher pH data.

**Figure 3:**
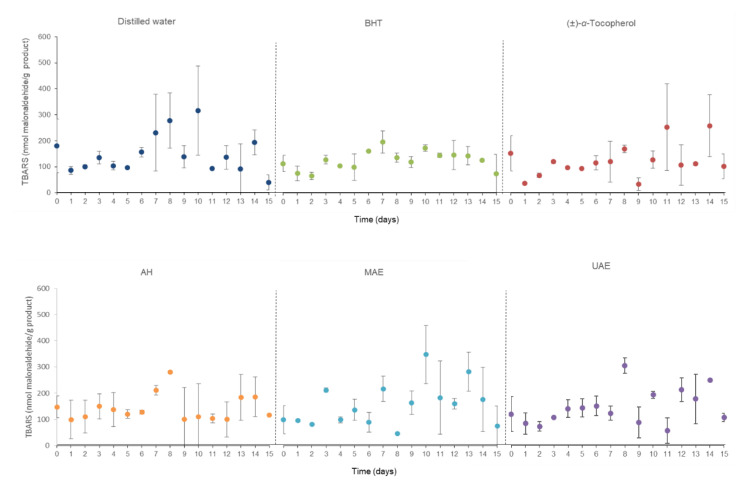
Thiobarbituric acid-reactive substance (TBARS) concentrations in accelerated oxidation assay at 50 °C during 15 days of the body milk samples elaborated with different *C. crispus* extracts. AH: Autohydrolysis; MAE: Microwave-assisted extraction; UAE: Ultrasound-assisted extraction; BHT: Butylated hydroxytoluene.

Figure 3 compiles the thiobarbituric acid-reactive substance (TBARS) concentrations of the body milk samples submitted to the acceleration oxidation assay to define the degree of lipid oxidations that took place in this procedure. In this case, these measurements oscillated around 300 units over time since the TBARS outcomes were in the range of approximately 35 to 348 nmol malonaldehyde/g personal care products. It should be noted that from Day 8 onwards, the fluctuations were generalized for all samples without a clear trend in all of them.

**Figure 4:**
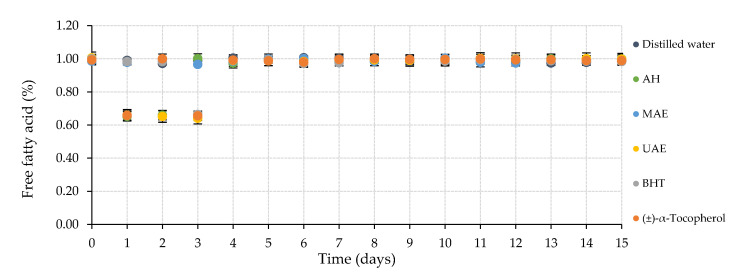
Compilation of free fatty acid (FFA, %) values during accelerated oxidation assay at 50 °C during 15 days of the body oil samples elaborated with different *C. crispus* extracts. AH: Autohydrolysis; MAE: Microwave-assisted extraction; UAE: Ultrasound-assisted extraction; BHT: Butylated hydroxytoluene.

Figure 4 indicates the impact of the utilized macroalgae extracts on the chemical properties of the studied body oil samples. Specifically, the free fatty acid (FFA) content was constant for the 15 days of treatment, as indicated by the plateau tendency of the obtained data, excluding the behavior described by the samples with AH and ultrasound-assisted extraction (UAE) extracts and butylated hydroxytoluene (BHT) and (±)-α-tocopherol commercial antioxidant compounds. These samples reduced their FFA percentage by about 0.4 units for Days 1 to 3, recovering their initial condition for the remaining days.

**Figure 5:**
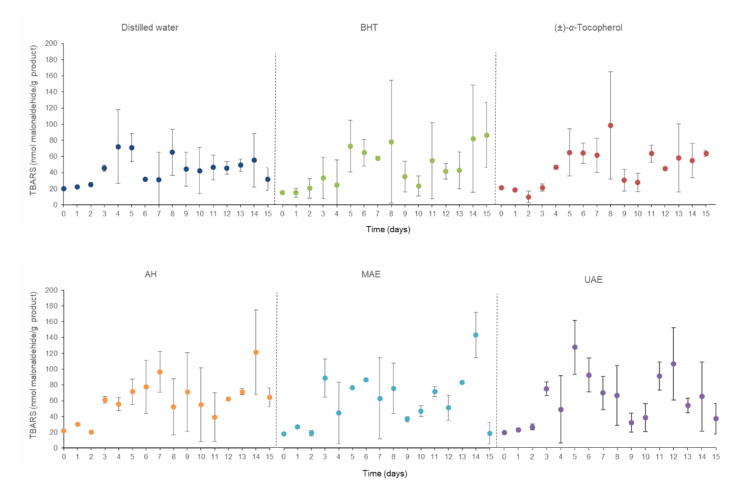
Thiobarbituric acid-reactive substance (TBARS) concentrations during accelerated oxidation assay at 50 °C during 15 days of the body oil samples elaborated with different *C. crispus* extracts. AH: Autohydrolysis; MAE: Microwave-assisted extraction; UAE: Ultrasound-assisted extraction; BHT: Butylated hydroxytoluene.

The TBARS profile of body oil samples (Figure 5) presented an opposed response since these values increased significantly from the second day, remaining stable up to this point (~29 nmol malonaldehyde/g personal care product). Hereafter, TBARS values were more noticeable, reaching the maximum level at about 143 nmol malonaldehyde/g personal care product to MAE samples at the final accelerated oxidation.

**Figure 6:**
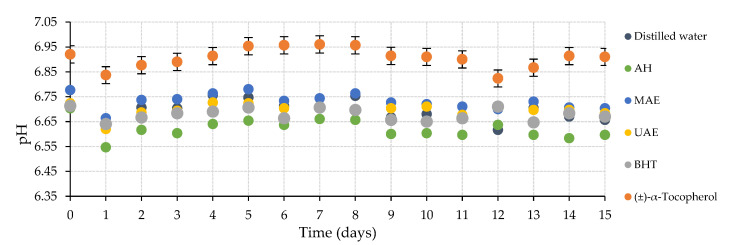
Compilation of pH values during accelerated oxidation assay at 50 °C during 15 days of the shampoo samples elaborated with different *C. crispus* extracts. AH: Autohydrolysis; MAE: Microwave-assisted extraction; UAE: Ultrasound-assisted extraction; BHT: Butylated hydroxytoluene.

Figure 6 recompiles the evaluated chemical characteristic of the tested shampoo samples. The plot displays the pH changes in these fresh cosmetic products, as well as these shampoos, after their thermal treatment. AH-enriched shampoo presented the lowest pH values (approximately 6.6) in contrast with samples with (±)-α-tocopherol since these kept the highest pH values throughout the whole process with an average of around 6.9. The pH data of the remaining samples were defined between around 6.6 and 6.8, so this light variation indicates that this parameter remained like the initial one.

**Figure 7:**
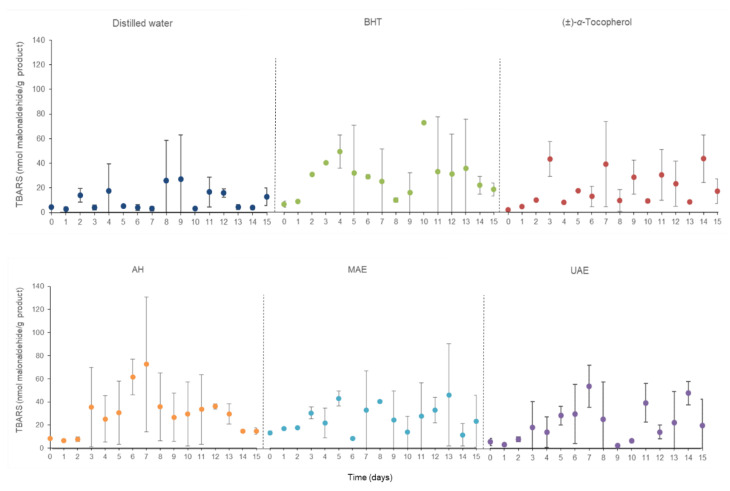
Thiobarbituric acid-reactive substance (TBARS) concentrations during accelerated oxidation assay at 50 °C during 15 days of the shampoo samples elaborated with different *C. crispus* extracts. AH: Autohydrolysis; MAE: Microwave-assisted extraction; UAE: Ultrasound-assisted extraction; BHT: Butylated hydroxytoluene.

In relation to the evolution of the TBARS concentration of the treated samples (Figure 7), it is noteworthy that the collected data did not show a clear trend. Although the initial and final TBARS concentrations were similar (about 6.8 in front of approximately 17.8 nmol malonaldehyde/g personal care product), the intermediate values reached average values of ~38.8 nmol malonaldehyde/g personal care product exposed by shampoo samples with AH *C. crispus* extract. These results suggest that the distinct added extracts and antioxidant compounds presented different effects on the development of secondary oxidation products in comparison with control shampoo elaborated with distilled water.

**Figure 8:**
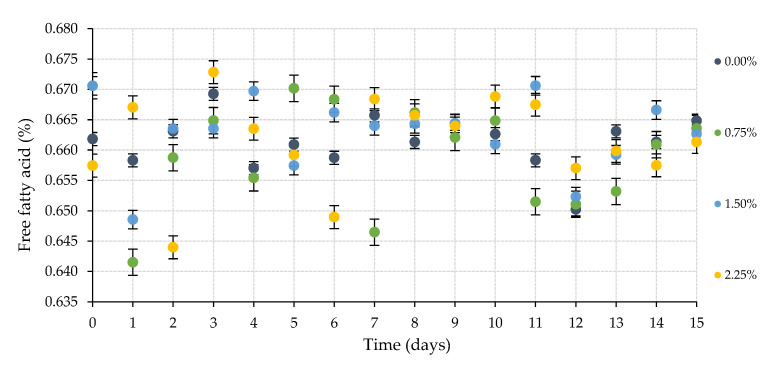
Compilation of free fatty acid (FFA, %) values during accelerated oxidation assay at 50 °C during 15 days of the body scrub samples elaborated with different percentages of dried macroalgae *C. crispus* by microwave hydrodiffusion and gravity technology (MHG).

Body scrub samples designed with some proportions of dried microwave hydrodiffusion and gravity technology (MHG) macroalgae were also subjected to stability analysis employing an accelerated oxidation assay for 15 days. The registered FFA values of these cosmetic models are represented in Figure 8.

**Figure 9:**
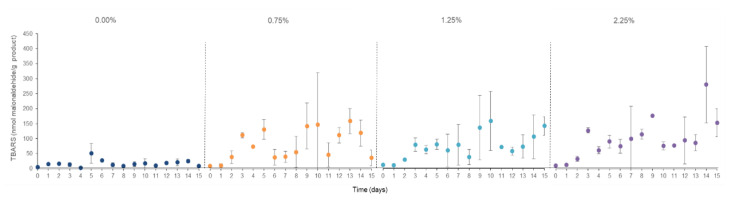
Thiobarbituric acid-reactive substance (TBARS) concentrations during accelerated oxidation assay at 50 °C during 15 days of the body scrub samples elaborated with different percentages of dried macroalgae *C. crispus* by microwave hydrodiffusion and gravity technology (MHG).

These results hardly varied by 0.03%; consequently, their FFA concentration can be considered as constant independently of the thermal and time treatment and the used macroalgae percentage. However, this last aspect disclosed is essential in the TBARS concentration of the studied cosmetic samples (Figure 9). In this case, a parallel rise of the generated malonaldehyde concentration was clearly linked with the quantity of the used dried algae. Further works on the storage stability of these personal care products should be conducted to extend the knowledge about the chemical reactions between the macroalgae’s bioactive compounds and the detected malonaldehyde amount.

Color coordinates and magnitudes of the body milk products are exhibited in Table 1 following the CIEL*a*b* method. The developed fresh cosmetics presented slight differences from each other since the range of the distinct coordinates was limited (L*, a* and b* coordinates fluctuated in the range from 85.27 to 85.43, from −4.14 to −4.80 and from 3.50 to 5.35, respectively). This aspect was supported by the small difference described by the total color difference (ΔE* < 1.5) between the studied enriched samples and the control product (elaborated with distilled water), except for the (±)-α-tocopherol sample because it showed a distinct color difference (1.5 < ΔE* < 3.0) in accordance with the classification established previously by Adekunte et al. [12]. On the other hand, Table 1 also displays the color data of the body milk samples treated at 50 °C for 15 days to define the effects of an accelerated oxidation assay. Body milk samples enriched with macroalgae extracts were hardly modified, because these samples kept their small difference to the control body milk, but samples obtained by the addition of commercial antioxidants (BHT and (±)-α-tocopherol) strongly increased the values of coordinates a* (more greenish color) and b* (more yellowish color), showing a total color difference of 6.90 and 3.93, respectively, vs. distilled water. The collected results comparing the values at 0 days and 15 days indicated that the total color difference (ΔE*) varied strongly with oxidation, being most marked in the samples with commercial antioxidant compounds, and the least change was recorded with the body milk enriched with the microwave extract.

Table 2 recompiles the color characterization of the tested body oil samples. These presented similar coordinates and magnitudes values independently of the employed extract or antioxidant, as well as the absence of the accelerated oxidation procedure or their application. The reduced average outputs of ΔE* parameters attested to it (around 0.25 and 0.08 for fresh body oil products and 0.38 and 0.27 for treated personal care products, respectively). The variation of the total color differences from 0 to 15 days ranged from 0.45 (UAE extract) to 1.01 (BHT), without a too appreciable perception of color change.

Color measurements of the elaborated shampoo samples with *C. crispus* extracts and commercial antioxidant compounds are displayed in Table 3. 

The samples that included macroalgae extracts from AH, MAE and UAE extraction technologies exhibited similar features, as shown by their magnitude values. For example, the hue angle (h*), chroma (C*) and saturation (S*) of average data of fresh samples were −15.85, 2.77 and 0.03, respectively. These samples defined a small total color difference in the Adekunte et al. ranking (i.e., ΔE* < 1.5) [12]. This tendency was also analyzed in the cosmetic products submitted to the accelerated oxidation process, and the average ΔE* value of the shampoos with macroalgae extracts was 1.54. Specifically, these outputs presented noticeably different to butylated hydroxytoluene (BHT) and (±)-α-tocopherol samples since ΔE* BHT values were around one-third higher than samples with macroalgae, extracts whereas that of the ΔE* (±)-α-tocopherol sample data was about eight times higher. The comparative ΔE* from 0 to 15 days of accelerated oxidation showed that samples fortified with the extracts obtained with AH or MAE showed a smaller color variation.

Table 4 shows the colorimetric features of the body scrub cosmetics elaborated with dried MHG *C. crispus* macroalgae. The results displayed an inverse relation with the added algae percentage and the values of lightness (L*) and coordinate b* since these values were reduced for fresh and heat-treated samples as the macroalgae percentage was intensified. Meanwhile, the coordinate a* described the opposed tendency, so its value grew simultaneously with the amount of used dried red macroalgae. A very distinct ΔE* was defined in all cases (0 and 15 days) since the obtained results exceeded the limit of 3.0 established by Adekunte et al. [12] in comparison with the control sample (0.00%), but the accelerated oxidation of each body scrub sample during 15 days of oxidation did not produce such marked differences in ΔE*, even in the sample added with 1.51% extract of dried *C. crispus* by MHG, and it was scarcely detectable (ΔE* = 0.88).

**Figure 10:**
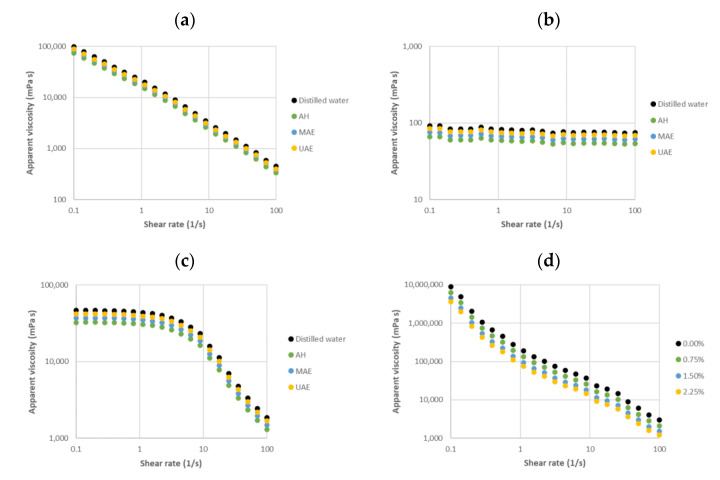
Viscosity profiles for (**a**) body milk, (**b**) body oil, (**c**) shampoo and (**d**) body scrub samples in the absence (black) and presence of selected *C. crispus* extracts: AH: Autohydrolysis; MAE: Microwave-assisted extraction; UAE: Ultrasound-assisted extraction. Note here that percentages in (**d**) correspond with the residual solids content obtained after MHG treatment.

Figure 10 shows the viscous profiles of the developed personal care products at 25 °C. Body milk samples exhibited shear-thinning behavior with a notable decrease (more than two decades) in the apparent viscosity with increasing shear rate (Figure 10a). In contrast, body oils presented Newtonian behavior, with the apparent viscosity almost invariant over the range of tested shear rates (Figure 10b). Shampoos showed intermediate profiles between body milks and oils, where the apparent viscosity featured a Newtonian plateau at the lowest shear rates (below 7.5 1/s) and then shear-thinning behavior (Figure 10c). Body scrub samples also exhibited a decrease in the apparent viscosity with increasing shear rate (Figure 10d). This type of personal care product presented the highest drop in the apparent viscosity (about four decades).

In all cases, the incorporation of selected extracts involved a slight weakness in the viscous behavior at a fixed shear rate when compared with systems prepared with distilled water in the absence of bioactive soluble extracts. Body milk, body oil and shampoos featured the highest impact on the apparent viscosity with the addition of extracts from *C. crispus* recovered by autohydrolysis, followed by those extracted from microwave-assisted extraction and ultrasound-assisted extraction. At the same shear rate, the apparent viscosity of body scrubs prepared with residual solids from the dehydration of *C. crispus* using microwave hydrodiffusion and gravidity decreased with increasing solids content. Note here that those prepared with the (±)-α-tocopherol and BHT standards featured similar magnitudes and tendencies. It should be highlighted that any tested system showed hysteresis effects. In addition, no water syneresis was observed in the prepared cosmetics after three months of both room temperature and cold storage. These are two important advantages from the manufacturing, storage and application point of view.

## 3. Discussion

The study of the cosmetic potential of red macroalgae *Chondrus crispus* extracts and dried macroalgae fractions as novel cosmetic ingredients was developed by the present research through their incorporation into end personal care products (e.g., body milk, body oil, shampoo and body scrub cosmetic models) and their physicochemical characterization. The obtained results suggest that this marine resource could provide different raw materials of relevant interest to the cosmetic industry due to its successful application in this specific marketplace subject.

Overall, the evaluation of the chemical features of the studied products disclosed that the addition of the target extracts and dried macroalgae into these prototype cosmetic products was satisfactory. For example, the utilization of the tested macroalgae extracts entailed a reduction in the pH value of body milk samples in comparison with this type of personal care product elaborated with distilled water as a control product. This effect can promote the preservation of the natural cutaneous condition, by the maintenance of the skin enzymatic activity and microbiota population, since the optimum acid profile of the skin surface presents a physiological protective character [13]. The acid feature was also shown in the shampoo samples, so they kept the agreement evidence established for topical cosmetic products [14]. The relationship between the pH of the designed personal care products and the skin surface pH after their topical application should be the object of further research to ensure the adequate skin health of future consumers. This parameter also has a notable influence on the preservation process of personal care products, among others [15]. Their monitoring by means of an accelerated oxidation assay at 50 °C for 15 days allowed recording the alterations produced during this process. In parallel, the stability of free fatty acid (FFA) values and thiobarbituric acid-reactive substance (TBARS) concentrations was also considered. Specifically, the evolution of this last parameter in the accelerated storage provided a general approach to the secondary lipid oxidation process that took place in the studied formulations, reflecting the potential use of the *C. crispus* extracts and dried samples as antioxidant elements in these cosmetic lipid-containing matrices. Their common tendency was similar to that obtained in other oxidative stability studies, in which the TBARS concentration increased until reaching a maximum limit and then decreased, defining a plateau region between these two points. Similar profiles were also shown by oil-in-water emulsions enriched with different concentrations of *Artemisia annua* [16] or *Arctostaphylos uva-ursi* leaf extracts [17].

On the other hand, the color determination of the tested cosmetic samples was also defined pre- and postrequired lipid oxidation procedure. The collected data showed comparable values for each personal care product, so the produced oxidative effects were barely influenced by their organoleptic features in accordance with Khanum and Thevanayagam’s indications [18]. This effect showed that reduced quality loss was likely produced on treated samples in the same way that the employment of *Euterpe oleracea* fruit extract could assist the preservation of the oil features during the storage of treated extra virgin olive oil samples in biodegradable active films [19]. However, in general, personal care products obtained by the addition of the *C. crispus* extracts showed a more stable color after forced oxidation than those in which commercial antioxidant compounds were added.

Rheological testing of the proposed natural personal care products exhibited profiles and magnitudes consistent with their synthetic commercial counterparts [10,20]. Those products supplemented with selected extracts suggested improved properties from the processing (related to the lowest shear rates) and applicability (related to the highest shear rates) points of view. This is an advantage from the industrial view, since the developed cosmetics with lower apparent viscosity over the tested shear rate could promote their spread on the skin [9,21]. According to this behavior, body milks, body oils and shampoos incorporated with extracts recovered using hydrothermal treatments could favor their spreadability on the skin and consequently the consumers’ acceptance. Namely, shampoo profiles indicated a solid structure at rest, whereas body cream featured a liquid structure at rest. This behavior is consistent with that reported for different biopolymer-based matrices in the absence of antioxidant fractions [22]. In the case of body scrubs, lower apparent viscosities at a fixed shear rate in the presence of a higher solid content from MHG seem to be related to an increase in competition for water available in the system [23]. This latter sample could spread out easier on the skin when compared with those prepared with a lower MHG solid content. Neither the hysteresis phenomenon nor water release was observed in the studied enriched personal care products with the consequent manufacturing and application advantages.

## 4. Materials and Methods

### 4.1. Personal Care Product Formulations

Four different cosmetic models were developed using the previously optimized extracts from red macroalgae *Chondrus crispus* by autohydrolysis (AH) [24], microwave-assisted extraction (MAE) [25] and ultrasound-assisted extraction (UAE) [26] technologies, as well as dried macroalgae by microwave hydrodiffusion and gravity (MHG) [21]. Table 5 summarizes the main processing conditions and extract features.

Body milk samples were elaborated incorporating tested extracts in a commercial organic body milk base (Gran Velada, S.L., Zaragoza, Spain) in a proportion of 0.5% (*w*/*w*), following the manufacturer’s instructions [27]. Note here that the amount of extract for the different formulations developed was based on previous work on personal care products formulated with natural extracts [21,27,28]. For this purpose, the body milk base was previously tempered at 40 °C using a water bath, and consecutively the extracts were added and properly homogenized.

Body oil products referred to the protocol of Soto et al. [28]. Specifically, almond oil (39.84%, *w*/*w*) (Guinama, S.L.U., La Pobla de Vallbona, Valencia, Spain), soya oil (39.84%, *w*/*w*) (Guinama, S.L.U., La Pobla de Vallbona, Valencia, Spain) and *Ricinus communis* oil (19.92%, *w*/*w*) (Guinama, S.L.U., La Pobla de Vallbona, Valencia, Spain) were mixed and homogenized at room temperature. Then, the evaluated extracts were added (0.40%, *w*/*w*) and properly integrated into the final product.

Shampoo samples were elaborated according to López-Hortas et al.’s methodology [29]. Specifically, a commercial shampoo base (Guinama, S.L.U., La Pobla de Vallbona, Valencia, Spain) after achieving a temperature of 40 °C was enriched with target extracts at a rate of 3.0% (*w*/*w*).

MHG dried *C. crispus* macroalgae following the conditions previously detailed [21] was added at different percentages (in the range of 0.00–2.25%) of body scrub formula based on Soto et al.’s [28] cosmetic formulation. The alga sample was previously ground by a domestic food processor to obtain a diameter particle size ≤1.0 mm. To elaborate these personal care products, sodium chloride (75.00–73.31%, *w*/*w*) (Guinama, S.L.U., La Pobla de Vallbona, Valencia, Spain) was mixed with almond oil (25.00–24.44%, *w*/*w*) (Guinama, S.L.U., La Pobla de Vallbona, Valencia, Spain) and the proportional macroalgae at room temperature until the homogenization of the cosmetic prototype.

*C. crispus* extracts were added to the cosmetic products due to their relevant antioxidant properties and replaced by distilled water to prepare control samples. In parallel, butylated hydroxytoluene (BHT) (Sigma-Aldrich, Missouri, MO, USA) and (±)-α-tocopherol (Sigma-Aldrich, Missouri, MO, USA) were also employed as references since these compounds are widely used as commercial antioxidants in this type of matrices. In all cases, the tested elaborations were prepared at least in duplicate, packed in sterile glass containers and stored in the light absence until their further analysis.

### 4.2. Physicochemical Analysis

#### 4.2.1. Accelerated Oxidative Determination

Freshly cosmetic products were characterized by the determination of their pH value (body milk and shampoo samples) or free fatty acid percentage (FFA, %) (body oil and scrub samples), as well as their thiobarbituric acid-reactive substance (TBARS) concentration. Specifically, the pH values of the cosmetic products were measured at room temperature using a pH meter GLP 21 (Hach Lange Spain, S.L.U., Spain) previously calibrated by standard solutions. FFA values were estimated by multiplying the factor 0.503, defined previously by Desta et al. [30], with the acid value of each sample. This determination was carried out following Atolani et al.’s indications [31] in such a way that the sample (0.5 g) and methanol (12.5 mL) with phenolphthalein indicator (1% solution in ethanol) were titrated with potassium hydroxide solution (0.1 M) at room temperature until the work solution changed to a pink appearance. Finally, TBARS were determined according to Scheffer et al.’s protocol [32] with slight adaptations. Samples (0.8 g) and thiobarbituric acid-butylated hydroxytoluene solution (1.6 mL) were mixed with a vortex for 30 s and rose to 95 °C for 15 min. After cooling of the samples in an ice-tap-water bath for 10 min and their degassing at 80 kHz 100% power for 15 min using an ultrasonic bath FB 11207 (Fisherbrand, Germany), the absorbance values were measured at 532 nm using an Evolution 201 ultraviolet-visible spectrophotometer (Thermo Fisher Scientific Inc., Waltham, Massachusetts, EE.UU.). These data were compared with those obtained using malonaldehyde solution as a standard pattern. All determinations were carried out at least in triplicate and were made in dark conditions

The physicochemical stability was also tested by an accelerated oxidation assay at 50 °C for 15 days, so these parameters above were defined daily in order to register the evolution of the produced changes in the cosmetic models’ examined values.

#### 4.2.2. Color Characterization

Color coordinates (L* (lightness, from 0 -whiteness degree- to 100 -brightness degree-); a* (red -a* > 0-/green -a* < 0- coordinate) and b* (yellow -b* > 0-/blue -b* < 0- coordinate) and magnitudes (hue angle (h*, °), chroma (C*) and saturation (S*)) of target personal care products were determined by CIEL*a*b* space employing a portable colorimeter CR-400 (Konica Minolta, Japan). The total color (ΔE*) differences (ΔE* = [(ΔL*)^2^ + (Δa*)^2^ + (Δb*)^2^]^1/2^) were also considered for comparative purposes. The measurements were made at least by quintuplicate to recently developed fresh samples (at 0 days), as well as to cosmetic models after being subjected at 50 °C for 15 days for their accelerated oxidation evaluation.

#### 4.2.3. Rheological Profiles

The viscous behavior of all formulated personal care products was assessed using steady-state shear measurements at least in duplicate. Rheological measurements were conducted on a controlled-stress rheometer (MCR 302, Paar Physica, Austria) by means of a sand-blasted plate–plate geometry to avoid slippage of the samples. The selected measured system was 25 mm in diameter with a 1 mm gap between plates, and it was controlled using a Peltier system (± 0.01 C). All flow curves were run at 25 °C. Samples were tempered at room temperature for 1 h before rheological testing. They were then placed on the measuring system, and the exposed edges were covered with light paraffin oil to prevent water losses during tests. In all cases, samples were rested 15 min before apparent viscosity measurements to favor thermal and structural equilibration.

### 4.3. Statistical Analysis

One-factor analysis of variance (ANOVA) was used to assess the significant differences between experimental data means. Post hoc analysis was performed using the well-known Scheffé test, which was selected to differentiate means with 95% confidence (*p* < 0.05). For this purpose, the software PASW Statistics v.22 (IBM SPSS Statistics, New York, NY, USA) was employed.

## Figures and Tables

**Table 1:** Comparison of the colorimetric characteristics by CIEL*a*b* system from the body milk samples elaborated with different *C. crispus* extracts. Values of the color coordinates and magnitudes of the fresh (at 0 days) and personal care product samples submitted to an accelerated oxidation assay at 50 °C for 15 days are summarized for comparative purposes.

**Parameter**	**Extracts (0 days)**					
	**Distilled** **Water**	**AH**	**MAE**	**UAE**	**BHT**	***α*-Tocopherol**
Lightness (L*)	85.42 ± 0.01 ^a^	85.27 ± 0.01 ^a^	85.35 ± 0.01 ^a^	85.43 ± 0.02 ^a^	85.37 ± 0.01 ^a^	85.40 ± 0.01 ^a^
Coordinate green-red (a*)	−4.17 ± 0.01 ^c^	−4.17 ± 0.01 ^c^	−4.14 ± 0.01 ^c^	−4.17 ± 0.02 ^c^	−4.46 ± 0.01 ^b^	−4.80 ± 0.01 ^a^
Coordinate blue-yellow (b*)	3.59 ± 0.02 ^d^	3.80 ± 0.01 ^c^	3.50 ± 0.02 ^d^	3.55 ± 0.05 ^d^	4.16 ± 0.01 ^b^	5.35 ± 0.00 ^a^
Hue angle (h*, °)	−40.70 ± 0.01 ^c^	−42.32 ± 0.01 ^b^	−40.22 ± 0.02 ^c^	−40.36 ± 0.02 ^c^	−43.01 ± 0.01 ^b^	−48.12 ± 0.01 ^a^
Chroma (C*)	5.50 ± 0.01 ^c^	5.64 ± 0.01 ^c^	5.43 ± 0.01 ^c^	5.48 ± 0.02 ^c^	6.10 ± 0.01 ^b^	7.19 ± 0.03 ^a^
Saturation (S*)	0.06 ± 0.01 ^a^	0.07 ± 0.01 ^a^	0.06 ± 0.01 ^a^	0.06 ± 0.01 ^a^	0.07 ± 0.01 ^a^	0.08 ± 0.01 ^a^
Total color difference (ΔE*) vs. distilled water	--	0.26	0.11	0.04	0.65	1.87
**Parameter**	**Extracts (15 days)**					
	**Distilled** **Water**	**AH**	**MAE**	**UAE**	**BHT**	***α*-Tocopherol**
Lightness (L*)	85.45 ± 0.01 ^a^	85.43 ± 0.01 ^a^	85.63 ± 0.01 ^a^	85.86 ± 0.01 ^a^	85.92 ± 0.01 ^a^	86.02 ± 0.01 ^a^
Coordinate green-red (a*)	−3.78 ± 0.01 ^c^	−3.93 ± 0.01 ^c^	−3.80 ± 0.01 ^c^	−3.85 ± 0.01 ^c^	−6.79 ± 0.01 ^a^	−5.22 ± 0.01 ^b^
Coordinate blue-yellow (b*)	4.75 ± 0.01 ^e^	5.62 ± 0.01 ^c^	4.39 ± 0.01 ^f^	5.15 ± 0.03 ^d^	10.94 ± 0.03 ^a^	8.37 ± 0.01 ^b^
Hue angle (h*, °)	−51.49 ± 0.01 ^d^	−55.01 ± 0.01 ^b^	−49.10 ± 0.01 ^e^	−53.21 ± 0.01 ^c^	−58.18 ± 0.02 ^a^	−58.06 ± 0.01 ^a^
Chroma (C*)	6.07 ± 0.01 ^e^	6.86 ± 0.01 ^c^	5.81 ± 0.01 ^f^	6.43 ± 0.01 ^d^	12.88 ± 0.02 ^a^	9.86 ± 0.01 ^b^
Saturation (S*)	0.07 ± 0.01 ^b^	0.08 ± 0.01 ^b^	0.07 ± 0.01 ^b^	0.07 ± 0.02 ^b^	0.15 ± 0.02 ^a^	0.11 ± 0.01 ^a^
Total color difference (ΔE*) vs. distilled water	--	0.88	0.40	0.58	6.90	3.93
**Parameter**	**Comparison 0 days versus 15 days**					
	**Distilled** **Water**	**AH**	**MAE**	**UAE**	**BHT**	***α*-Tocopherol**
Total color difference (ΔE*)	1.22	1.84	0.99	1.69	7.19	3.11

Data are given as mean ± standard deviation. Data values in a row with different letters are statistically different (*p* ≤ 0.05). AH: Autohydrolysis; MAE: Microwave-assisted extraction; UAE: Ultrasound-assisted extraction; BHT: Butylated hydroxytoluene

**Table 2:** Comparison of the colorimetric characteristics by CIEL*a*b* system from the body oil samples elaborated with different *C. crispus* extracts. Values of the color coordinates and magnitudes of the fresh (at 0 days) and personal care product samples submitted to an accelerated oxidation assay at 50 °C for 15 days are summarized for comparative purposes.

**Parameter**	**Extracts (0 days)**					
	**Distilled** **Water**	**AH**	**MAE**	**UAE**	**BHT**	***α*-Tocopherol**
Lightness (L*)	90.32 ± 0.01 ^a^	90.26 ± 0.01 ^a^	90.42 ± 0.01 ^a^	90.15 ± 0.01 ^a^	89.82 ± 0.01 ^b^	90.61 ± 0.01 ^a^
Coordinate green-red (a*)	−2.96 ± 0.01 ^a^	−2.95 ± 0.01 ^a^	−2.92 ± 0.01 ^a^	−2.94 ± 0.01 ^a^	−3.00 ± 0.01 ^a^	−3.01 ± 0.03 ^a^
Coordinate blue-yellow (b*)	1.22 ± 0.01 ^b^	1.25 ± 0.01 ^b^	1.30 ± 0.01 ^a,b^	1.19 ± 0.00 ^b^	1.31 ± 0.02 ^a^	1.43 ± 0.08 ^a^
Hue angle (h*, °)	−22.32 ± 0.01 ^e^	−23.00 ± 0.01 ^d^	−24.08 ± 0.01 ^b^	−22.01 ± 0.01 ^e^	−23.54 ± 0.01 ^c^	−25.44 ± 0.01 ^a^
Chroma (C*)	3.20 ± 0.01 ^a^	3.21 ± 0.01 ^a^	3.19 ± 0.01 ^a^	3.17 ± 0.01 ^a^	3.27 ± 0.01 ^a^	3.33 ± 0.03 ^a^
Saturation (S*)	0.04 ± 0.01 ^a^	0.04 ± 0.01 ^a^	0.04 ± 0.01 ^a^	0.04 ± 0.01 ^a^	0.04 ± 0.01 ^a^	0.04 ± 0.01 ^a^
Total color difference (ΔE*) vs. distilled water	--	0.07	0.14	0.18	0.51	0.37
**Parameter**	**Extracts (15 days)**					
	**Distilled** **Water**	**AH**	**MAE**	**UAE**	**BHT**	***α*-Tocopherol**
Lightness (L*)	90.56 ± 0.01 ^a^	90.44 ± 0.01 ^a^	90.43 ± 0.01 ^a^	90.56 ± 0.01 ^a^	90.41 ± 0.01 ^a^	90.74 ± 0.01 ^a^
Coordinate green-red (a*)	−2.64 ± 0.02 ^a^	−2.67 ± 0.01 ^a^	−2.82 ± 0.01 ^a^	−2.75 ± 0.01 ^a^	−2.60 ± 0.02 ^a^	−2.72 ± 0.01 ^a^
Coordinate blue-yellow (b*)	0.59 ± 0.01 ^c^	0.70 ± 0.01 ^b^	1.24 ± 0.01 ^a^	1.21 ± 0.01 ^a^	0.60 ± 0.01 ^c^	0.76 ± 0.01 ^b^
Hue angle (h*, °)	−12.60 ± 0.01 ^c^	−14.61 ± 0.01 ^b^	−23.76 ± 0.01 ^a^	−23.78± 0.01 ^a^	−12.91 ± 0.01 ^c^	−15.69 ± 0.01 ^b^
Chroma (C*)	2.71 ± 0.01 ^b^	2.76 ± 0.01 ^b^	3.08 ± 0.01 ^a^	3.00 ± 0.01 ^a^	2.67 ± 0.01 ^b^	2.82 ± 0.01 ^a^
Saturation (S*)	0.03 ± 0.01 ^a^	0.03 ± 0.01 ^a^	0.03 ± 0.01 ^a^	0.03 ± 0.01 ^a^	0.03 ± 0.01 ^a^	0.03 ± 0.01 ^a^
Total color difference (ΔE*) vs. distilled water	--	0.16	0.68	0.63	0.15	0.26
**Parameter**	**Comparison 0 days versus 15 days**					
	**Distilled** **Water**	**AH**	**MAE**	**UAE**	**BHT**	***α*-Tocopherol**
Total color difference (ΔE*)	0.75	0.64	0.95	0.45	1.01	0.74

Data are given as mean ± standard deviation. Data values in a row with different letters are statistically different (*p* ≤ 0.05). AH: Autohydrolysis; MAE: Microwave-assisted extraction; UAE: Ultrasound-assisted extraction; BHT: Butylated hydroxytoluene

**Table 3:** Comparison of the colorimetric characteristics by CIEL*a*b* system from the shampoo samples elaborated with different *C. crispus* extracts. Values of the color coordinates and magnitudes of the fresh (at 0 days) and personal care product samples submitted to an accelerated oxidation assay at 50 °C for 15 days are summarized for comparative purposes.

**Parameter**	**Extracts (0 days)**					
	**Distilled** **Water**	**AH**	**MAE**	**UAE**	**BHT**	***α*-Tocopherol**
Lightness (L*)	89.28 ± 0.01 ^b^	89.82 ± 0.01 ^b^	90.86 ± 0.01 ^a^	90.26 ± 0.01 ^a^	84.20 ± 0.01 ^c^	81.91 ± 0.01 ^d^
Coordinate green-red (a*)	−2.72 ± 0.01 ^a^	−2.83 ± 0.02 ^a^	−2.56 ± 0.01 ^b^	−2.58 ± 0.01 ^b^	−2.48 ± 0.01 ^b^	−2.76 ± 0.03 ^a^
Coordinate blue-yellow (b*)	0.20 ± 0.01 ^e^	1.06 ± 0.01 ^b^	0.81 ± 0.01 ^c^	0.44 ± 0.01 ^d^	0.96 ± 0.01 ^b^	2.08 ± 0.01 ^a^
Hue angle (h*, °)	−4.14 ± 0.01 ^e^	−20.45± 0.01 ^b^	−17.47 ± 0.01 ^c^	−9.62 ± 0.01 ^d^	−21.09 ± 0.01 ^b^	−37.04 ± 0.01 ^a^
Chroma (C*)	2.72 ± 0.01 ^c^	3.02 ± 0.01 ^b^	2.69 ± 0.01 ^c^	2.61 ± 0.01 ^c^	2.66 ± 0.01 ^c^	3.45 ± 0.02 ^a^
Saturation (S*)	0.03 ± 0.01 ^a^	0.03 ± 0.01 ^a^	0.03 ± 0.01 ^a^	0.03 ± 0.01 ^a^	0.03 ± 0.01 ^a^	0.04 ± 0.01 ^a^
Total color difference (ΔE*) vs. distilled water	--	1.02	1.69	1.01	5.15	7.61
**Parameter**	**Extracts (15 days)**					
	**Distilled** **Water**	**AH**	**MAE**	**UAE**	**BHT**	***α*-Tocopherol**
Lightness (L*)	89.22 ± 0.01 ^b^	90.02 ± 0.01 ^a^	89.89 ± 0.01 ^a^	89.01 ± 0.00 ^b^	84.57 ± 0.01 ^c^	80.63 ± 0.02 ^d^
Coordinate green-red (a*)	−0.67 ± 0.03 ^d^	−2.18 ± 0.01 ^b^	−2.02 ± 0.02 ^b,c^	−1.93 ± 0.01 ^c^	−1.93 ± 0.01 ^c^	−2.69 ± 0.02 ^a^
Coordinate blue-yellow (b*)	−0.16 ± 0.00 ^e^	0.35 ± 0.00 ^b^	−0.01 ± 0.00 ^d^	−0.39 ± 0.01 ^f^	0.10 ± 0.01 ^c^	8.23 ± 0.03 ^a^
Hue angle (h*, °)	13.50 ± 0.01 ^b^	−9.11 ± 0.01 ^f^	0.28 ± 0.01 ^d^	11.31 ± 0.01 ^c^	−3.06 ± 0.01 ^e^	−71.91 ± 0.01 ^a^
Chroma (C*)	0.69 ± 0.01 ^c^	2.21 ± 0.01 ^b^	2.02 ± 0.01 ^b^	1.97 ± 0.01 ^b^	1.94 ± 0.01 ^b^	8.66 ± 0.01 ^a^
Saturation (S*)	0.01 ± 0.01 ^b^	0.02 ± 0.01 ^b^	0.02 ± 0.01 ^b^	0.02 ± 0.01 ^b^	0.02 ± 0.01 ^b^	0.11 ± 0.01 ^a^
Total color difference (ΔE*) vs. distilled water	--	1.79	1.52	1.30	4.83	12.18
**Parameter**	**Comparison 0 days versus 15 days**					
	**Distilled** **Water**	**AH**	**MAE**	**UAE**	**BHT**	***α*-Tocopherol**
Total color difference (ΔE*)	2.08	0.98	1.38	1.64	1.09	6.28

Data are given as mean ± standard deviation. Data values in a row with different letters are statistically different (*p* ≤ 0.05). AH: Autohydrolysis; MAE: Microwave-assisted extraction; UAE: Ultrasound-assisted extraction; BHT: Butylated hydroxytoluene

**Table 4:** Comparison of the colorimetric characteristics by CIEL*a*b* system from the body scrub samples elaborated with different percentages of dried macroalgae *C. crispus* by MHG technology. Values of the color coordinates and magnitudes of the fresh (at 0 days) and personal care product samples submitted to an accelerated oxidation assay at 50 °C for 15 days are summarized for comparative purposes.

**Parameter**	**Extracts (0 days)**			
	**0.00%**	**0.75%**	**1.50%**	**2.25%**
Lightness (L*)	76.41 ± 0.02 ^a^	61.56 ± 0.10 ^b^	57.74 ± 0.01 ^c^	50.83 ± 0.02 ^d^
Coordinate green-red (a*)	−4.21 ± 0.01 ^a^	−2.44 ± 0.02 ^b^	−2.05 ± 0.02 ^c^	−1.15 ± 0.01 ^d^
Coordinate blue-yellow (b*)	4.46 ± 0.01 ^c^	5.35 ± 0.02 ^b^	6.80 ± 0.01 ^a^	6.92 ± 0.01 ^a^
Hue angle (h*, °)	−46.61 ± 0.21 ^d^	−65.44 ± 0.08 ^c^	−73.19 ± 0.01 ^b^	−80.56 ± 0.01 ^a^
Chroma (C*)	6.13 ± 0.01 ^b^	5.88 ± 0.01 ^c^	7.10 ± 0.01 ^a^	7.01 ± 0.01 ^a^
Saturation (S*)	0.08 ± 0.01 ^b^	0.10 ± 0.01 ^a,b^	0.12 ± 0.01 ^a^	0.14 ± 0.01 ^a^
Total color difference (ΔE*) vs. distilled water	--	14.98	18.95	25.89
**Parameter**	**Extracts (15 days)**			
	**0.00%**	**0.75%**	**1.50%**	**2.25%**
Lightness (L*)	71.03 ± 0.02 ^a^	63.97 ± 0.01 ^b^	58.19 ± 0.01 ^c^	57.54 ± 0.01 ^c^
Coordinate green-red (a*)	−3.38 ± 0.01 ^a^	−2.63 ± 0.01 ^b^	−1.62 ± 0.03 ^c^	−1.57 ± 0.03 ^c^
Coordinate blue-yellow (b*)	3.30 ± 0.01 ^d^	6.73 ± 0.02 ^c^	7.42 ± 0.04 ^b^	7.89 ± 0.02 ^a^
Hue angle (h*, °)	−44.31 ± 0.01 ^c^	−68.66 ± 0.01 ^b^	−77.70 ± 0.03 ^a^	−78.73 ± 0.01 ^a^
Chroma (C*)	4.72 ± 0.01 ^c^	7.23 ± 0.01 ^b^	7.59 ± 0.02 ^b^	8.05 ± 0.01 ^a^
Saturation (S*)	0.07 ± 0.01 ^c^	0.11 ± 0.01 ^b^	0.13 ± 0.01 ^a^	0.14 ± 0.01 ^a^
Total color difference (ΔE*) vs. distilled water	--	7.89	13.61	14.37
**Parameter**	**Comparison 0 days versus 15 days**			
	**0.00%**	**0.75%**	**1.50%**	**2.25%**
Total color difference (ΔE*)	5.57	2.78	0.88	6.79

Data are given as mean ± standard deviation. Data values in a row with different letters are statistically different (*p* ≤ 0.05).

**Table 5:** Processing conditions and fundamental composition of the extracts used for the personal care products development.

Extracts	AH [24]	MAE [25]	UAE [26]	MHG [21]
Extraction conditions (T, t, S, SLR)	200 °C, -, W, 1:30	170 °C, 3 min, W, 1:30	80 °C, 3 min, W, 1.5:100	800 W, 2 min and 100, W, 30 min
Yield (%)	89.73 ± 0.12	55.62 ± 0.95	44.30 ± 0.35	28.00 ± 0.54
GAE content (mg/g)	1.85 ± 0.01	9.21 ± 0.12	12.98	-
TEAC (mg/g)	3.91 ± 0.02	85.10 ± 1.83	178	-
Protein content (mg/g)	7.72 ± 0.03	22.43 ± 2.41	21–22	13.1 ± 0.4
Carbohydrate content (%)	39.51 ± 0.21	43.30 ± 0.61	-	54.6 ± 1.5
Ash content (%)		-	-	24.4 ± 0.1

T: Processing temperature; t: Processing time; S: Extractive agent; SLR: Solid–liquid ratio; W: Water; GAE: Gallic acid equivalents; TEAC: Trolox equivalent antioxidant capacity.

## Data Availability

Data is contained within the article.
